# The effect of somatosensory input on motor imagery depends upon motor imagery capability

**DOI:** 10.3389/fpsyg.2015.00104

**Published:** 2015-02-12

**Authors:** Nobuaki Mizuguchi, Takahiro Yamagishi, Hiroki Nakata, Kazuyuki Kanosue

**Affiliations:** Faculty of Sport Sciences, Waseda University, Tokorozawa, Saitama, Japan

**Keywords:** mental practice, tactile, tennis, mental chronometry, tool-use

## Abstract

We investigated that the relationship between motor imagery ability and the effect of tactile input associated with holding a tennis racket on motor imagery of the forehand and backhand swings. The effect was assessed by the time utilized for motor imagery (mental chronometry). Seventeen tennis players imagined forehand and backhand swings with a forehand grip, a backhand grip or while holding nothing. In all cases, imaging the swings took longer than the time taken for a real swing. For imagery of the backhand swing, holding a racket with a backhand grip decreased the imaging time (*p* < 0.05) as compared to the trials with a forehand grip or while holding nothing. On the other hand, holding the racket with a backhand grip tended to increase the time required for forehand swing imagery. These results suggest that a congruent grip improves, and an incongruent grip deteriorates, motor imagery of the backhand swing. For players who took a longer time in the condition where they held nothing (i.e., poor imaging ability), the effect of a congruent backhand grip was greater (*r* = 0.67, *p* < 0.01). However, a congruent forehand grip did not improve motor imagery of the forehand swing. Since 15 of the participants in the present study favored the forehand swing compared to the backhand swing, the participants would have been more familiar with the forehand swing. Thus it would have been easy to vividly imagine the (familiar) forehand swing even when they were not holding a racket. We speculate that tactile input associated with holding a tool improves a vividness of motor imagery of a less familiar movement, especially for those who have poor imaging ability. In the future, it will be important to clarify whether the effect of tactile input associated with holding a tool is dependent upon movement familiarity/performance level.

## INTRODUCTION

Motor imagery is defined as the mental execution of an action without any overt movement or muscle activation (Jeannerod, 2001). Many previous studies have demonstrated that motor imagery training (mental practice) improves motor skills (Feltz and Landers, 1983; Pascual-Leone et al., 1995; Allami et al., 2008). Therefore, this training is widely used in sports as well as for recovery of function following motor impairment (Lotze and Halsband, 2006; Mizuguchi et al., 2012a). However, the efficacy of motor imagery training is dependent upon an individual’s imaging ability (Isaac, 1992). That is, participants with a high motor imaging ability, as assessed by a vividness of imagery questionnaire, show a greater performance improvement following motor imagery training than do participants with a low imaging ability.

Neurophysiological studies utilizing functional magnetic resonance imaging (fMRI) or transcranial magnetic stimulation (TMS) have shown that brain activity during motor imagery is similar to the activation that is seen during motor execution (Kasai et al., 1997; Hashimoto and Rothwell, 1999; Hanakawa et al., 2003, 2008; Lacourse et al., 2005; Lotze and Halsband, 2006; Imazu et al., 2007; Guillot et al., 2009; Mizuguchi et al., 2013b). For example, the supplemental motor area, premotor area, and parietal cortices were activated during motor imagery (Hanakawa et al., 2003, 2008; Imazu et al., 2007; Guillot et al., 2009; Mizuguchi et al., 2013b). The primary somatosensory cortex (S1) was also activated during motor imagery even when somatosensory signals generated by touching a tool or an object were absent (Gao et al., 2011; Mizuguchi et al., 2013a). These results suggest that not only motor related regions but also somatosensory regions play an important role in the creation of motor imagery.

Our previous studies utilizing TMS and fMRI revealed that appropriate tactile input generated by holding a relevant object increased brain activity during motor imagery of very simple movements such as grasping and pinching (Mizuguchi et al., 2009, 2011, 2012b, 2013a). This evidence indicates that tactile input improves the quality of motor imagery. Recent studies suggest that appropriate tactile input generated by holding a tool improves the quality of the motor imagery of complex movements such as a tennis swing (Bisio et al., 2014; Wang et al., 2014). These results imply that motor imagery training for sports skills that involve the utilization of a tool, such as a bat or racket, would be more effective if the training was done while holding the tool. These studies also showed that the effect of tactile input on motor imagery of complex movements was greater for athletes than naïve participants (Bisio et al., 2014; Wang et al., 2014). Thus, the effect of tactile input on motor imagery might depend on the participants’ movement experience, as well as their abilities in sensorimotor representation and/or motor imagery ability. Motor imagery ability differs across individuals, and certainly also across athletes. However, it remains unclear as to whether the effect of tactile input on motor imagery differs to an important degree across among athletes.

In the present study, we sought to clarify the relationship between motor imagery ability in athletes and the effect of tactile input associated with holding a tool on motor imagery for complex movements. To this end we calculated the correlation coefficient between motor imagery ability and the effect of tactile input associated with holding a tool on the quality of motor imagery. We hypothesized that effects of tactile input associated with holding a tool would be greater for participants with a lower imagery ability because the participants with a higher imagery ability would likely utilize their sensorimotor representation. That is, for the participants with the higher imagery ability, the external somatosensory information might not be needed so much during motor imagery. To evaluate motor imagery ability, we employed the mental chronometry method (Sirigu et al., 1996; Guillot and Collet, 2005; Gentili et al., 2010; Bisio et al., 2014; Wang et al., 2014). We utilized forehand and backhand tennis swings as representative of complex sport movements. We utilized tennis players to compare the time spent performing actual tennis swings with the time spent for motor imagery of the same swings. If the duration of the actual movement and the motor imagery were similar, it would indicate a high level of motor imagery quality (Guillot and Collet, 2005). We also investigated the effect of congruent and incongruent grips on the imagery of the swing, since most tennis players use a different grip for their forehand and backhand swings. To clarify the relationship between motor imagery ability and the effect of tactile input associated with holding a tool may contribute to sports training or rehabilitation after stroke.

## EXPERIMENTAL PROCEDURES

### PARTICIPANTS

Seventeen tennis players (five females and 12 males, age: range 19–29 years old, mean 22.1 ± 2.3 years old) participated in this study. They all had at least 2 years of experience playing tennis (range 2–12 years, mean 7.1 ± 3.0 years). All Participants received a detailed explanation of the experimental procedures before the experiment, and written informed consent was obtained from all participants. The study was approved by the Human Research Ethics committee of Waseda University.

### PROCEDURE

Participants performed under eight different task conditions: actual forehand swings while holding a racket with the forehand grip (FS); actual backhand swings while holding a racket with the backhand grip (BS); imagery of a forehand swing while holding a racket with the forehand grip (FIF); imagery of a forehand swing while holding a racket with the backhand grip (FIB); imagery of a forehand swing while not holding a racket (FIN); imagery of a backhand swing while holding a racket with the forehand grip (BIF); imagery of a backhand swing while holding a racket with the backhand grip (BIB); and imagery of a backhand swing while not holding a racket (BIN). The participants were asked to perform 10 consecutive shadow swings in the FS and BS conditions, and similarly to imagine 10 consecutive swings in the FIF, FIB, FIN, BIF, BIB, and BIN conditions. We chose to require 10 consecutive swings rather than just one swing because the time for single swing would make an accurate analysis difficult and, among other things increase the variability of the response time. Five trials were performed for each condition (i.e., 40 trials in total). To avoid fatigue, the participants took a 5-min break after the first 20 trials. The order of the eight conditions was randomized. Before the experiment began, each participant was given an explanation about the difference between the first person and third person perspectives (Stevens, 2005). The subjects were subsequently instructed to “imagine a forehand or backhand swing with the first person perspective.” An initial unrecorded practice session involving several trials was conducted in order to familiarize the participants with the conditions of the experiment.

**FIGURE 1:**
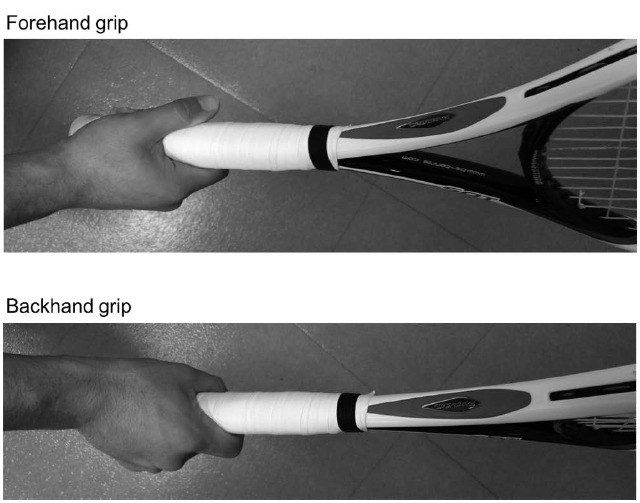
**Grips of forehand and backhand swings for a representative participant**.

The tasks took place in a quiet room. The participant executed shadow swings with a racket, or sat in a chair and imagined swings with their eyes closed (Wang et al., 2014). The participants used their own rackets, and swung using their normal forehand and backhand grips (Figure 1). To measure the time of execution for both real and imagined tasks, the participants were asked to press a switch with their foot at the start and end of execution for both the real and imagined swings (Gentili et al., 2010). The signals were digitized via an A/D converter system at 1,000 Hz and stored for later analysis.

To confirm the effect of the tactile input that derives holding a tool on the subject’s subjective vividness, we assessed the vividness of motor imagery after the six task conditions that involved imagery. For this we utilized a 10-point Likert scale (10 = vivid imagery, 1 = not vivid imagery; Lebon et al., 2012). In addition, the participants were asked about whether they preferred using the forehand or backhand swing.

### DATA ANALYSIS

To reduce the effect of individual differences in the actual swings, the duration of trials in the FIF, FIB, and FIN conditions were normalized to the average duration of the FS trials. Those in the BIF, BIB, and BIN conditions were normalized to the average duration of the BS trials. To evaluate the effect of tactile input associated with holding the racket, differences between the normalized durations among the imagery conditions were tested by a two-way analysis of variance (ANOVA) with repeated measures using the within-subject factors of swing (forehand and backhand), and grip (forehand grip, backhand grip, and not-hold). If a violation of the sphericity assumption occurred when we ran Mauchly’s test, the Greenhouse–Geisser epsilon correction coefficient was used to correct the degrees of freedom; *F* and *P* values were then recalculated. *Post hoc* analyses were performed utilizing paired *t*-tests with the Bonferroni correction for dependent samples.

To investigate the relationship between motor imagery ability and the effect of tactile input associated with holding a racket, a Pearson’s correlation coefficient between “the normalized duration in not-holding condition” and “the difference in duration between the conditions of not-holding and holding with the grip” was calculated.

The scores across the questionnaires for the different conditions were tested by the Friedman test. *Post hoc* analyses were determined utilizing Wilcoxon signed-rank tests with the Bonferroni correction. All tests were evaluated utilizing a 95% confidence interval. Data values were expressed as the mean ± one standard error (SE).

## RESULTS

**FIGURE 2:**
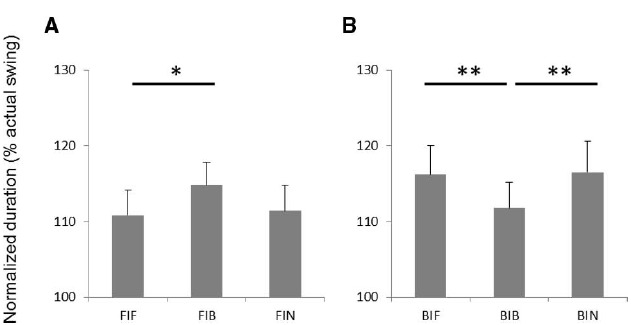
**The durations of imaginary 10 consecutive swings for (A) forehand swings and (B) backhand swings.** FIF, forehand swing imagery with holding a racket with backhand grip condition; FIB, forehand swing imagery with holding a racket with backhand grip condition; FIN, forehand swing imagery without holding; BIF, backhand swing imagery with holding a racket with holding forehand grip condition; BIB, backhand swing imagery with holding a racket with backhand grip condition; BIN, backhand swing imagery without holding condition. *p < 0.05, **p < 0.01.

Average durations of actual and imagined swings are shown in Table 1. Normalized durations of imagined forehand swing and backhand swing for all conditions are shown in Figures 2A,B. There was no significant main effect for the factors of swing and grip. However, a swing-grip interaction was found [*F*(2,32) = 11.86, *p* < 0.01, ${\text{η}}_{\text{p}}^{\text{2}}$ = 0.43]. *Post hoc* tests for forehand swing imagery showed that the duration was significantly longer in condition FIB than in FIF (*p* < 0.05; Figure 2A). This indicates that the time spent for forehand swing imagery with a backhand grip was increased as compared to that with a forehand grip. In addition, the duration tended to be greater in condition FIB than in FIN (*p* = 0.069, uncorrected; Figure 2A). For imagery of the backhand swing, the duration of imagery was significantly shorter in the BIB condition than in BIF or BIN (*p* < 0.01, respectively; Figure 2B). This indicates that the time spent for backhand swing imagery with a backhand grip decreased as compared to that with the forehand grip or while not holding a racket.

**Table 1:** **Average durations for the different conditions**.

Forehand swings (seconds)				Backhand swings (seconds)			
FS	FIF	FIB	FIN	BS	BIF	BIB	BIN
22.2 ± 1.1	24.6 ± 1.4	25.5 ± 1.5	24.7 ± 1.4	22.0 ± 1.3	25.4 ± 1.6	24.4 ± 1.4	25.5 ± 1.6

**FIGURE 3:**
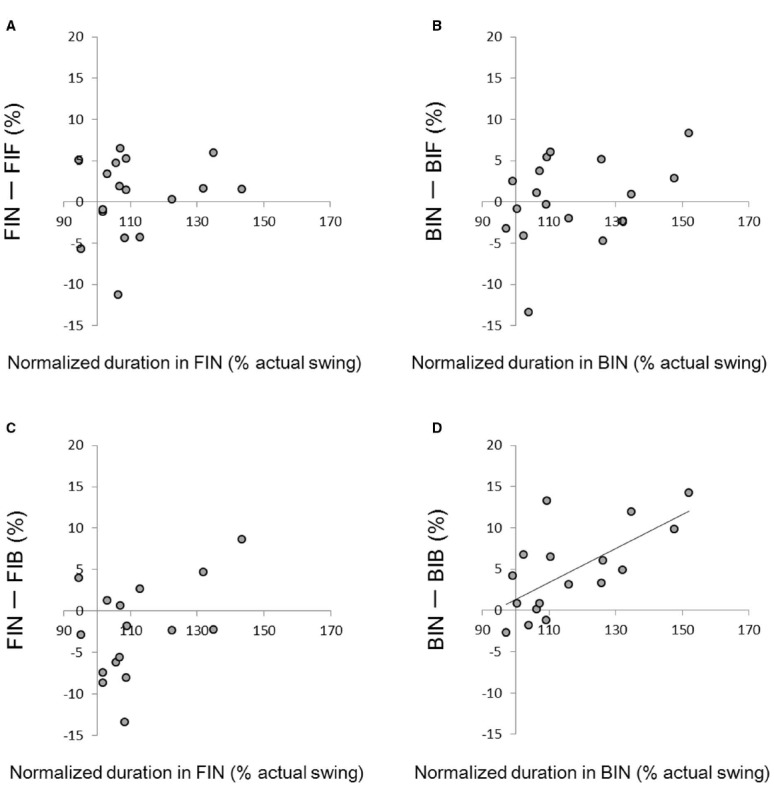
**(A)** Relationship between the normalized time difference in FIN and the difference between FIN and FIF; **(B)** the normalized time difference in BIN and the difference between BIN and BIF; **(C)** the normalized time difference in FIN and the difference between FIN and FIF; **(D)** the normalized time difference in BIN and the difference between BIN and BIB (*r* = 0.67, *p* < 0.01).

In order to analyze how holding a racket influenced the duration of swing imagery, changes in duration between trials with and without holding a racket were plotted against the duration without holding a racket (Figure 3). A significant correlation was obtained between the normalized duration in the BIN condition and the difference in duration between BIB and BIN (*r* = 0.67, *p* < 0.01; Figure 3D). This indicates that the effect of a backhand grip was larger for participants who took longer to complete the imagery. We did not find any correlations in the other three graphs.

**FIGURE 4:**
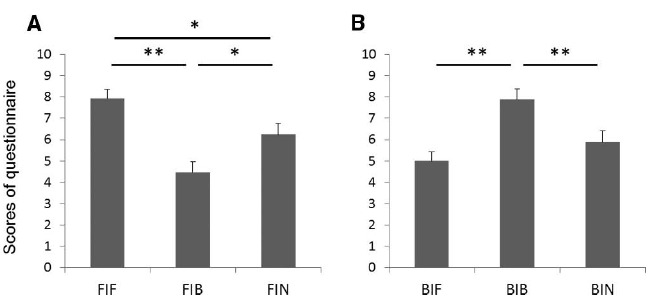
**Scores of the questionnaire for (A) forehand swings and (B) backhand swings (10 = vivid imagery, 1 = not vivid imagery).** *p < 0.05, **p < 0.01.

The mean participant scores of the questionnaire questions on vividness of imagery are shown in Figure 4. A Friedman test demonstrated a significant difference across conditions (*p* < 0.01). *Post hoc* tests for forehand swing imagery showed that the score was significantly larger (more vivid) in the FIF condition than in FIB and FIN (*p* < 0.01 and *p* < 0.05, respectively; Figure 4A). In addition, the score was significantly larger in the FIN condition than in FIB (*p* < 0.05). For backhand swing imagery, the score was larger in the BIB condition than in BIF or BIN (*p* < 0.01 for both; Figure 4B). This result indicates that congruent grips improved the subjective vividness of tennis swings.

Fifteen of the 17 participants preferred a forehand swing, while the remaining two preferred a backhand swing. These preferences were consistent with the results on mental chronometry.

## DISCUSSION

We utilized mental chronometry to investigate the effect of holding a racket on imagery of the tennis swing in tennis players (Sirigu et al., 1996; Guillot and Collet, 2005; Gentili et al., 2010; Bisio et al., 2014; Wang et al., 2014). All imagery tasks, including those involving both the forehand and backhand, took longer than an actual swing. This is consistent with the result of a previous study which showed that the duration of imaging a tennis swing takes longer than an actual tennis swing (Guillot and Collet, 2005; Bisio et al., 2014; Wang et al., 2014). In the present study, motor imagery ability was evaluated by the difference between the duration of the actual swing while holding a racket and the imagery duration when the participants did not hold a racket (FIN and BIN for the forehand and backhand swing, respectively).

For imagery tasks involving the backhand swing, the duration of imagery was significantly shorter in the BIB condition, in which the swing and grip type matched, than the BIF, in which they did not match, and the BIN, in which no racket was held (Figure 3B). Previous studies show that the smaller the difference between the duration of actual movement and the duration of motor imagery, the more vivid the motor imagery (Sirigu et al., 1996; Guillot and Collet, 2005). In addition, in our questionnaire (which was completed after all the imagery tasks) the score of vividness was significantly larger in the BIB condition than in the BIF and BIN conditions (Figure 4B). Thus, a congruent backhand grip enabled participants to produce a more vivid imagery of the backhand swing than did the incongruent forehand grip or holding nothing.

The effect of holding a racket with a congruent backhand grip was greater for those participants with a lower imaging ability. That is, for those participants who took a longer time for imaging when they were not holding a racket (Figure 4D). This would imply that for those with a lower imaging ability, holding a tool or object in an appropriate way could be helpful for creating a more vivid motor imagery. In other words, it is likely that people with good imaging ability would be able to have vivid imagines even without the somatosensory signals generated by holding the relevant tool or an object. Previous studies using fMRI report a significant activation of the S1 during motor imagery (Gao et al., 2011; Mizuguchi et al., 2013a). This S1 activity might be related to efference copy from the motor cortices (Grush, 2004). It has been proposed that such efference copy, which would be generated together with motor command, is used to compare the peripheral sensory signal with the expected sensory signal (Anderson and Lenz, 2011). Therefore, S1 activity during motor imagery might reflect the expected sensory signal which could be created from the efference copy of the imagined movement. We speculate that those people who have a good imaging ability could refer to their sensorimotor representation and thus compensate for a lack of tactile input related to holding a tool during motor imagery. That is, they might be able to generate a more correct or vivid sensorimotor representation that is close to the afferent signal they would have obtained by actually holding a racket. This would explain why motor imagery training or mental practice can improve motor skills without the somatosensory signals or error information that is present when real movements are executed.

If the congruency of swing grip type improved the quality of tennis swing imagery, the duration of the FIF condition (forehand swing with forehand grip) should have been shorter that the FIN (forehand swing with no racket). However, in the present study, there was no difference in duration between FIF and FIN. Most participants preferred the forehand swing over the backhand swing because the forehand swing is easier to acquire and more powerful than the backhand. Indeed, the forehand swing is generally favored by both beginners and advanced players, and is used more frequently in both games and practice. Thus, it was probably easier for our participants to vividly imagine the familiar forehand swing and/or anticipate the somatosensory signals caused by a forehand grip even when they were not holding a racket. This being so, actually holding a racket with a forehand (congruent) grip would then not be likely to further improve the quality of forehand swing imagery (ceiling effect). However, we did not specifically evaluate their familiarity or motor performance relative to the different grips. In the future, it will be important to clarify whether the effect of tactile input associated with holding a tool is dependent upon movement familiarity/performance level. In addition, in order to clarify whether the forehand grip would improve the quality of forehand imagery in novice players, it would be useful to collect data from tennis players with less experience than those of the present study. Furthermore, it would be interesting to investigate why a different effect was observed between imageries of the forehand and backhand swings while holding the racket by measuring brain activity in association with the anticipated somatosensory signals during both imageries.

When the participants held the racket with an incongruent backhand grip, the forehand imagery became less vivid. However, holding the racket with a forehand grip did not influence imagery for the backhand swing. Since the participants would have been familiar with the forehand swing, the incongruent tactile information might have deteriorated the motor imagery of the familiar movement. Taking the results of the imaging of the forehand and backhand swings together, we speculate that the positive effect of congruent tactile input for motor imagery was greater for unfamiliar movements or for participants with poor imagery. However, the negative effect of incongruent tactile input would be greater for familiar movements. Previous studies suggest that holding a tool has no effect on naïve participants (Bisio et al., 2014; Wang et al., 2014). For tool holding to improve the quality of motor imagery, it is thus necessary to have experience with the tool-use or its sensorimotor representation. Therefore, holding a tool during motor imagery might be more useful for players at an intermediate stage, and for movements that are not fully acquired yet. As mentioned above, further study will be needed to clarify this hypothesis.

A limitation of the present study was that the participants imagined 10 consecutive swings, while previous studies used only a single swing (Guillot and Collet, 2005; Bisio et al., 2014; Wang et al., 2014). Since a tennis swing is an acyclical movement, its imagery might be different for imagery of a single acyclical movement and the 10 consecutive acyclical movements that we utilized. However, our results were consistent with those of previous studies (Guillot and Collet, 2005; Bisio et al., 2014; Wang et al., 2014). Therefore, we believe that our results obtained with the imagery of repetitive acyclical movements are likely to be similar to those that would have been obtained with a single acyclical movement.

## CONCLUSION

Our results showed that, for relatively experienced tennis players, the duration of motor imagery of tennis swings was modified depending upon the congruency/incongruency of racket grip and swing type. Holding the racket in an appropriate way during motor imagery improved the quality of the imagery. This effect was stronger for the backhand swing than for the normally preferred forehand swing.

### Conflict of Interest Statement

The authors declare that the research was conducted in the absence of any commercial or financial relationships that could be construed as a potential conflict of interest.
